# Supplementary material for: “We are left with nothing to work with”; challenges of nurses working in the emergency unit at a secondary referral hospital: A descriptive qualitative study
Source: PLoS One. 2021 Feb 18;16(2):e0247062. doi: 10.1371/journal.pone.0247062 (PMC7891734; doi:10.1371/journal.pone.0247062)
Supplement: S1 File — (DOCX) [file pone.0247062.s001.docx]

| **MIAN THEMES** | **EXAMPLE QUOTES** |
| --- | --- |
| **Overcrowding in the emergency unit** | *“Currently the bed capacity in the emergency unit is only two but you can sometimes have as many as five to eight patients at the same time and some may end up sitting down or we use worn-out stretchers as improvised beds that are not comfortable for them to receive their treatments. The overcrowding caused by limited beds and space makes it very difficult for us to move between patients to deliver care. We are at risk because anything can happen to us as caregivers.”* (Participant 129)  *“…at times, we can have fifteen patients at once, and we have two beds in the emergency room and there are times that we have to nurse patients in chairs…and this makes work very difficult”* (Participant 127)  *“…the stretchers, wheelchairs with the beds make the place so congested…the environment is very small and the way many patients are rushed in makes the place not conducive”* (Participant 125) |
| **Understaffing at the emergency unit** | *“In terms of staff strength, we are lacking a lot to the extent that sometimes only one staff works in the emergency unit per shift”* (Participant 212)  *”…there is no trained emergency nurse in the unit. Staffing challenges make work very tedious because, work that four (4) or five (5) people should do, it is left to one person. This makes nursing care very difficult and leads to exhaustion. This leads to attention deficit and inadequate care for the client. Our emergency is very busy, we work 24/7 and you could manage about twenty patients during the day shift and I am always very tired when I get home”* (Participant 127) |
| **Lack of emergency equipment** | *“…We have two BP [blood pressure] apparatuses that we share with the general Out Patient Department [OPD], so when a case comes to the emergency unit, you have to run to the OPD for the BP apparatus and the general OPD is on hold or chasing you back for the same apparatus. And the patient looks at you, that, you don’t know what you are doing”* (Participant 127)  *“Sometimes unavailability of emergency drugs brings a lot of problems in the emergency unit…we are left with nothing to work with…”* (Participant 212)  *“… sometimes patient report to the emergency and both the hospital and emergency stock is finished and we have to use our own money to buy common intravenous fluid [IVF] giving set. There are no glucometer strips to even check the patient’s random blood sugar [RBS] when they come in collapsed. You either run to the wards for glucometer strips or send a relative to buy them outside. We sometimes don’t have gloves or syringes to work with and have to use our bare hands for most of the invasive procedures. You have situations where there is no oxygen in the whole hospital and you are found wanting in front of a client who needs oxygen...”* (Participant 311)  *“… sometimes common disposable gloves are not available as well as safety boxes and it exposes us to a lot of occupational risks. You will run everywhere for oxygen just because there is no oxygen in the hospital and at the end of the day, you lose your client”* (Participant 129) |
| **Inadequate managerial support** | *“It’s very difficult to get our superiors to understand what we are facing in the emergency unit. Immediately they come and the place is busy, you will hear them say, ‘this place is heavy ooo, and they will disappear’. I wish that they will come, and work with us and understand what we have been going through so that they won’t be saying that we are always complaining. This challenge is always with the nursing administration because the medical superintendent is always with us and he understands what we go through. For our nursing administration, they always say we talk or complain too much, and it has got to a time where everybody says they will do what they can, and this is affecting the quality of care given to client and job satisfaction”* (Participant 127)  *“…nursing administration doesn’t understand the plight of the nurses working in the emergency unit. When we complain to them about consumables and logistics, they will shout at you saying that you complain too much. Meanwhile, the client is in front of you and you can’t circumvent the problem. They like using this phrase- ‘you’re misusing the things’. Nursing administration doesn’t give us listening ears and even if they do, they will say - ‘during my time it was like this’. We do what we can because our managers are not ready to support us. They tag you when you are trying to push for the best interest of the client”* (Participant 311)  *“Over the years nobody had recognized me for the works I’ve been doing- not even a handshake. If you do a good job, you need to be recognized and appreciated but that never happens but they’re very quick to chastise you for a little mistake done. This doesn’t motivate me to give my all”* (Participant 121)  *“…I’m not aware of any policy of the hospital concerning the upgrading of nurses and nurses working at the emergency unit. Sometimes colleague nurses may do presentations on conditions and whoever is asked to do this presentation, research, and come and present. This is not effective; we need specialist training in emergency care nursing…”* (Participant 129) |
