# Supplementary material for: “We are left with nothing to work with”; challenges of nurses working in the emergency unit at a secondary referral hospital: A descriptive qualitative study
Source: PLoS One. 2021 Feb 18;16(2):e0247062. doi: 10.1371/journal.pone.0247062 (PMC7891734; doi:10.1371/journal.pone.0247062)
Supplement: Attachment — Submitted filename: PONE-D-20-22261_reviewer TM.pdf [file pone.0247062.s002.pdf]

PLOS ONE

“We are left with nothing to work with”; challenges of nurses working in the emergency unit at a secondary referral hospital: a descriptive qualitative study

--Manuscript Draft--

**Manuscript Number:**	PONE-D-20-22261
**Article Type:**	Research Article
**Full Title:**	“We are left with nothing to work with”; challenges of nurses working in the emergency unit at a secondary referral hospital: a descriptive qualitative study
**Short Title:**	challenges of nurses working in the emergency unit at a secondary referral hospital
**Corresponding Author:**	Agani Afaya<br>University of Health and Allied Sciences<br>Ho, GHANA
**Keywords:**	Emergency Nursing, Emergency Department, Challenges
**Abstract:**	Introduction<br><br>In recent times, there has been an increasing burden in traumatic, medical, and surgical emergency conditions, placing more emphasis on the need for quality emergency care. This study aimed to explore the challenges experienced by nurses working in the emergency unit of a secondary referral hospital.<br><br>Methods<br><br>The study used an exploratory qualitative research design with a constructivist approach and a grounded theory method. Data were collected through semi-structured in-depth interviews lasting between 30 to 45 minutes using an interview guide. Inductive content analysis was used to analyse data because there were no pre-empted themes that were used to guide the study.<br><br>Results<br><br>Eleven (11) participants were interviewed and the majority were female (9), aged between 31-40 years. From the inductive content analysis, four themes emerged from the data and were; 1) overcrowding in the emergency unit, 2) understaffing at the emergency unit, 3) lack of emergency equipment, 4) Inadequate managerial support<br><br>Conclusion<br><br>The study identified several major challenges confronting nurses working in the emergency which are linked with managerial processes and inadequate managerial support. These challenges need to be addressed to promote quality emergency nursing care delivery. It is important for the government, and hospital management to invest attention and resources into emergency departments to reduce the situation of lack of resources for the emergency team to work with. Management that validates the expressions of concern from ED nurses will foster a positive working environment.
**Order of Authors:**	Agani Afaya
	Victoria Bam
	Thomas Bavo Azongo
	Richard Adongo Afaya
	George Kwame Kpodo
	Robert Alhassan Kaba
	Denis Albanus Nangsire Zinle
	Daniel Kofi Tayuu

Powered by Editorial Manager® and ProduXion Manager® from Aries Systems Corporation

	Stella Asantewaa
	Peter Adatara
**Additional Information:**	
**Question**	**Response**
**Financial Disclosure**<br><br>Enter a financial disclosure statement that describes the sources of funding for the work included in this submission. Review the submission guidelines for detailed requirements. View published research articles from *PLOS ONE* for specific examples.<br><br>This statement is required for submission and **will appear in the published article** if the submission is accepted. Please make sure it is accurate.<br><br>**Unfunded studies**<br>Enter: *The author(s) received no specific funding for this work.*<br><br>**Funded studies**<br>Enter a statement with the following details:<br>• Initials of the authors who received each award<br>• Grant numbers awarded to each author<br>• The full name of each funder<br>• URL of each funder website<br>• Did the sponsors or funders play any role in the study design, data collection and analysis, decision to publish, or preparation of the manuscript?<br>• **NO** - Include this sentence at the end of your statement: *The funders had no role in study design, data collection and analysis, decision to publish, or preparation of the manuscript.*<br>• **YES** - Specify the role(s) played.<br><br>* typeset	The author(s) received no specific funding for this work.
**Competing Interests**<br><br>Use the instructions below to enter a competing interest statement for this submission. On behalf of all authors, disclose any competing interests that could be perceived to bias this work—acknowledging all financial support and any other relevant financial or non-	The authors have declared that no competing interests exist.

financial competing interests.<br><br>This statement **will appear in the published article** if the submission is accepted. Please make sure it is accurate. View published research articles from *PLOS ONE* for specific examples.<br><br>**NO authors have competing interests**<br><br>Enter: *The authors have declared that no competing interests exist.*<br><br>**Authors with competing interests**<br><br>Enter competing interest details beginning with this statement:<br><br>*I have read the journal's policy and the authors of this manuscript have the following competing interests: [insert competing interests here]*<br><br>* typeset	
**Ethics Statement**<br><br>Enter an ethics statement for this submission. This statement is required if the study involved:<br><br>• Human participants<br>• Human specimens or tissue<br>• Vertebrate animals or cephalopods<br>• Vertebrate embryos or tissues<br>• Field research<br><br>Write "N/A" if the submission does not require an ethics statement.<br><br>General guidance is provided below. Consult the submission guidelines for detailed instructions. **Make sure that all information entered here is included in the Methods section of the manuscript.**	Ethics approval to conduct this study was obtained from the University of Health and Allied Sciences Research Ethics Committee (UHAS-REC A.9 [5] 18-19) Ho, Ghana. Permission was sought from the hospital management and unit manager before the commencement of data collection. Participants were informed about the purpose of the study and those that consented to participate in the study were included. Participants were informed about their right to withdraw from the study at any particular point in time. Confidentiality, privacy, and anonymity were assured. Random codes were given to participants to maintain anonymity.

Powered by Editorial Manager® and ProduXion Manager® from Aries Systems Corporation

**Format for specific study types**<br><br>**Human Subject Research (involving human participants and/or tissue)**<br>• Give the name of the institutional review board or ethics committee that approved the study<br>• Include the approval number and/or a statement indicating approval of this research<br>• Indicate the form of consent obtained (written/oral) or the reason that consent was not obtained (e.g. the data were analyzed anonymously)<br><br>**Animal Research (involving vertebrate animals, embryos or tissues)**<br>• Provide the name of the Institutional Animal Care and Use Committee (IACUC) or other relevant ethics board that reviewed the study protocol, and indicate whether they approved this research or granted a formal waiver of ethical approval<br>• Include an approval number if one was obtained<br>• If the study involved *non-human primates*, add *additional details* about animal welfare and steps taken to ameliorate suffering<br>• If anesthesia, euthanasia, or any kind of animal sacrifice is part of the study, include briefly which substances and/or methods were applied<br><br>**Field Research**<br><br>Include the following details if this study involves the collection of plant, animal, or other materials from a natural setting:<br>• Field permit number<br>• Name of the institution or relevant body that granted permission	
**Data Availability**<br><br>Authors are required to make all data underlying the findings described fully available, without restriction, and from the time of publication. PLOS allows rare exceptions to address legal and ethical concerns. See the PLOS Data Policy and FAQ for detailed information.	Yes - all data are fully available without restriction

Powered by Editorial Manager® and ProduXion Manager® from Aries Systems Corporation

A Data Availability Statement describing where the data can be found is required at submission. Your answers to this question constitute the Data Availability Statement and **will be published in the article**, if accepted.<br><br>**Important:** Stating 'data available on request from the author' is not sufficient. If your data are only available upon request, select 'No' for the first question and explain your exceptional situation in the text box.<br><br>Do the authors confirm that all data underlying the findings described in their manuscript are fully available without restriction?	
**Describe where the data may be found in full sentences. If you are copying our sample text, replace any instances of XXX with the appropriate details.**<br><br>• If the data are **held or will be held in a public repository**, include URLs, accession numbers or DOIs. If this information will only be available after acceptance, indicate this by ticking the box below. For example: *All XXX files are available from the XXX database (accession number(s) XXX, XXX.)*.<br>• If the data are all contained **within the manuscript and/or Supporting Information files**, enter the following: *All relevant data are within the manuscript and its Supporting Information files.*<br>• If neither of these applies but you are able to provide **details of access elsewhere**, with or without limitations, please do so. For example:<br><br>*Data cannot be shared publicly because of [XXX]. Data are available from the XXX Institutional Data Access / Ethics Committee (contact via XXX) for researchers who meet the criteria for access to confidential data.*<br><br>*The data underlying the results presented in the study are available from (include the name of the third party*	All relevant data are within the manuscript and its Supporting Information files.

and contact information or URL).<br>• This text is appropriate if the data are owned by a third party and authors do not have permission to share the data.<br><br>* typeset	
Additional data availability information:	

Manuscript
Click here to access/download;Manuscript;Manuscript.docx

Manuscript title: “**We are left with nothing to work with”; challenges of nurses working in the emergency unit at a secondary referral hospital: a descriptive qualitative study**

Agani Afaya[1*] (PhD student), Victoria Bam[2] (PhD), Thomas Bavo Azongo[3] (PhD), Richard Adongo Afaya[2] (MPhil), George Kwame Kpodo[1] (BN), Robert Alhassan Kaba[4] (PhD), Denis Albanus Nangsire Zinle[1] (BN), Daniel Kofi Tayuu[1] (BN), Stella Asantewaa[1] (BN), Peter Adatara (PhD)

[1]Department of Nursing, School of Nursing and Midwifery, University of Health and Allied Sciences Ho, Ghana
[2]Department of Nursing, Kwame Nkrumah University of Science and Technology, Kumasi, Ghana
[3]Department of Public Health, School of Allied Health Sciences, University for Development Studies, Tamale, Ghana
[4]Centre for Health Policy and Implementation Research. Institute of Health Research University of Health and Allied Sciences, Ho, Ghana

Correspondence
Agani Afaya (aagani@uhas.edu.gh)
School of Nursing and Midwifery, University of Health and Allied Sciences, Ho, Ghana

Abstract

Introduction: In recent times, there has been an increasing burden in traumatic, medical, and surgical emergency conditions, placing more emphasis on the need for quality emergency care. This study aimed to explore the challenges experienced by nurses working in the emergency unit of a secondary referral hospital.

Methods: The study used an exploratory qualitative research design with a constructivist approach and a grounded theory method**.** Data were collected through semi-structured in-depth interviews lasting between 30 to 45 minutes using an interview guide. Inductive content analysis was used to analyse data because there were no pre-empted themes that were used to guide the study.

Results: Eleven (11) participants were interviewed and the majority were female (9), aged between 31-40 years. From the inductive content analysis, four themes emerged from the data and were; 1) overcrowding in the emergency unit, 2) understaffing at the emergency unit, 3) lack of emergency equipment, 4) Inadequate managerial support

Conclusion: The study identified several major challenges confronting nurses working in the emergency which are linked with managerial processes and inadequate managerial support. These challenges need to be addressed to promote quality emergency nursing care delivery. It is important for the government, and hospital management to invest attention and resources into emergency departments to reduce the situation of lack of resources for the emergency team to work with. Management that validates the expressions of concern from ED nurses will foster a positive working environment.

Introduction

Emergency care is an important component of every health system. In recent times, there has been an increasing burden in traumatic, medical, and surgical emergency conditions across the globe, placing more emphasis on the need for quality emergency care [1]. The World Health Organization (WHO) identified sub-Saharan Africa (SSA) with 1.3% of the world's health care workers and 25% of the global disease burden [2]. Unfortunately, this burden of emergency care is high in low-resource countries compared with the developed world, with developing countries having deficiencies in organisational planning, shortage of trained healthcare personnel and resources to treat emergency conditions [1, 3, 4, 5]. It is estimated that more than 90% of injury-related deaths occur in low-resource countries [6] where the burden of injury in SSA represents a major public health threat and highlights the need for skilled health care personnel in the emergency departments to help avert preventable deaths.

Emergency care is important for the immediate management and stabilisation of critically ill patients. Although long overlooked as an essential component of healthcare in SSA, emergency health services are now growing across many countries. Emergency nursing is a specialty within professional nursing whereby the nurse provides care for patients requiring immediate medical attention to avoid long-term disability or death [2]. The emergency nurse plays a critical role in the identification and management of patients with life-threatening conditions, prioritizes emergency care through triaging, performs resuscitation with appropriate management within a supportive health care setting [2, 7]. The emergency nurse also provides information and emotional support to families or relatives of the ill person [7].

Due to the shortage of nursing staff, limited specialty training, overwhelming patient volumes, and high stressful working environments, the emergency nursing role especially in Africa is particularly challenging [8, 9].

In the provision of quality health care across Africa, nurses play a central role. Therefore, it is imperative to increase the skilled nursing workforce and provide specialty emergency nursing training across the sub-region. In response to these, the international community has made efforts in low and middle-income countries to develop capacity and quality improvement programs for emergency care [1, 10] and Ghana is not an exception. Most recent studies have called for increased attention to trauma and emergency care, but the health sector in Ghana currently has limited training institutions for these areas, resulting in delays in emergency care, and associated with poor clinical outcomes [7]. In 2012, Ghana started a degree programme in emergency nursing to help breach the gap of lack of specialist emergency care nurses in the country. Since the introduction of the Emergency nursing programme in Ghana, there has been an increase in the number of emergency nurses in the country, however, most emergency units in hospitals across the country continue to have inadequate or no specialist emergency care nurses. Studies conducted within some emergency centres in Ghana identified some challenges confronting nurses working in the ED [11]. It is therefore prudent to explore the challenges of ED nurses in order to provide recommendations to improve the emergency care system in Ghana. The key to responding to these challenges in emergency practice is research that produces discoveries, knowledge, and solutions that will contribute to providing safe and high-quality emergency nursing care. This study aimed to explore the challenges experienced by nurses working in the emergency unit of a secondary referral hospital.

Methods

Study design

The study used an exploratory qualitative research design with a constructivist grounded theory approach [12]. An interpretative approach [13] was employed to gain an in-depth understanding of the challenges that the nurses face during emergency care delivery. A grounded theory method was used to provide explicit, sequential guidelines for conducting this qualitative research, which offered specific strategies for handling the analytic phase of inquiry, streamlined and integrated data collection, and analysis with an advanced conceptual analysis of data [12]. In undertaking a constructivist survey the researchers adopted a position of mutuality where there was a relationship with participants that enables a mutual construction of meaning during interviews and a meaningful reconstruction of their stories into a grounded theory model [12, 14].

Study setting and population

This research was conducted at the emergency unit of a secondary referral hospital in the Ho municipality. It is one of the Ghana Health Services' (GHS) facilities situated in the heart of the Volta Regional Capital, Ho. The facility provides healthcare services to people living within the Ho Municipality and beyond. The hospital has a total bed capacity of one hundred and forty (140). The emergency unit is a small unit attached to the outpatient department (OPD) that runs twenty-four-hour emergency care services to clients. It admits and detains patients for not more than twenty-four hours. The emergency unit had two beds. The unit also utilized a couch and five stretchers as beds.

The participants for this study were registered general nurses and clinical nurse assistants with an active professional identification number (PIN) and auxiliary identification number (AIN) respectively who have been working in the emergency unit for a period not less than six (6) months.

At the time of the study, the emergency unit had no emergency nurse specialist, there were 2 senior nursing officers, a nursing officer, a senior staff nurse, 4 staff nurses, and 4 clinical nurse assistants working in the unit. The nurses in the ED had an eight-hour shift system for day duty while the night duty went for 12 hours.

Sampling and data collection procedure

Participants for the study were selected through purposive sampling method. In qualitative research, purposeful sampling is a technique that is widely used for the identification and selection of information-rich cases for the most effective use of limited resources [15]. This involves identifying and selecting participants that are available and willing to participate and should be able to communicate their experiences and opinions in an expressive, articulate, and reflective manner [16, 17, 18]. Data were collected through in-depth semi-structured interviews lasting up to 30 to 45 minutes using an interview guide. Face to face individual interviews was conducted at the emergency unit of the study hospital in a secluded office. Due to the busy nature of the unit, the authors had to arrange a convenient time with participants that were willing to participate in the study. Questions were asked about emergency care delivery and the challenges experienced during care delivery in the unit. Prompt and probing questions were used for further information and explanation following participants' responses. Each interview session was carried out by AA., and GK. During the interview sessions, the researchers ensured a conducive atmosphere where participants expressed themselves freely without interferences, and leading questions were avoided to ensure that participants' experiences remained intact. Data saturation was reached on the eleventh participant when no new information was obtained. All interviews were recorded by a digital sound recorder and transcribed verbatim by GKK, DANZ, DKT, and SA. During interviews some field notes were taken about the atmosphere, and some personal observations were made

afterward about the ward environment. All audio files and transcripts were stored anonymously in a secured protected digital storage system.

Data analysis

The study employed qualitative content analysis to analyse data as described by Padgett [19]. The appropriate form of analysis was inductive content analysis because there were no pre-empted themes to guide the study. The researchers read and reread the transcripts several times to make meaning of participants' views/challenges. Transcripts were coded after reading word by word and individual sentences carefully to identify words or phrases that spelled out the meaning of the sentences. During the coding process and before the start of coding AA and GKK coded the same interviews to identify and discuss the differences in coding to check for intercoder reliability. Similar codes were categorized to form themes and to ensure that they were representative of participants' views, the researchers met and discussed thoroughly the themes and agreed on the themes after several deliberations.

Trustworthiness

To ensure the trustworthiness, the researchers applied the principles of credibility, dependability, confirmability, and transferability [20]. Member checks were done at the end of each interview to ensure participants' views were well presented ensuring credibility. The researchers provided a detailed description of the study setting, methodology (COREQ criteria were used) [21], and background of the study sample to allow for the transferability of the findings in a similar context and setting. To ensure confirmability, an audit trail was kept for other researchers to validate the processes undertaken in the study. Dependability of findings was tested by re-reading the data to determine the depth of evidence available for each of the final themes and subthemes analysed by the research team.

Ethical consideration

Ethics approval to conduct this study was obtained from the University of Health and Allied Sciences Research Ethics Committee **(UHAS-REC A.9 [5] 18-19)** Ho, Ghana**.** Permission was sought from the hospital management and unit manager before the commencement of data collection. Participants were informed about the purpose of the study and those that consented to participate in the study were included. Participants were informed about their right to withdraw from the study at any particular point in time. Confidentiality, privacy, and anonymity were assured. Random codes were given to participants to maintain anonymity.

Results

Socio-Demographic Characteristics of Participants

A total of eleven (11) participants were interviewed including 8 registered general nurses and 3 clinical nurse assistants. The majority of the participants were women (9) and aged between 31-40 years. Most of the participants (7) had more than 3 years of working experience in the emergency unit (table 1).

Table 1: Socio-Demographic Characteristics of Participants

Characteristics		Number
Total		11
**Gender**	Male	2
	Female	9
**Age**	21-30	2
	31-40	9
**Marital Status**	Single	4

	Married	7
**Academic Qualification**	Certificate	3
	Diploma	5
	Bachelors	3
**Nursing Qualification**	Clinical Nurse Assistant	3
	Registered General Nurse	8
**Working Experience**	6months-3years	3
	4years-7years	6
	>7years	2

From the inductive content analysis, four themes emerged from the data which were; 1) overcrowding in the emergency unit, 2) understaffing at the emergency unit, 3) lack of emergency equipment, 4) inadequate managerial support.

***Theme 1;* Overcrowding in the emergency unit**

Overcrowding in the emergency unit was a great concern for participants. Participants expressed that inadequate space in the unit hinders quality nursing care delivery. Participants described the emergency unit as a ‘chamber’ meaning it is too small to be used as an emergency ward. Some participants also described the ward as very hot due to poor ventilation.

A participant narrated

> *“Currently the bed capacity in the emergency unit is only two but you can sometimes have as much as five to eight patients at the same time and some may end up sitting down or we use worn-out stretchers as improvised beds which are not comfortable for them to receive their treatments. The overcrowding caused by limited bed and space makes it very difficult*

for us to move between patients to deliver care. We are at risk because anything can happen to us as caregivers.” (Participant 129)

Almost all the participants expressed their frustration in having to nurse patients on wheelchairs and stretchers making the performance of some nursing procedures practically impossible. Participants reported not performing the full iterative circle of the nursing process due to unconducive working environment. Maneuvering through defective stretchers, wheelchairs, and benches in the delivery of quality emergency care are frustrating and pose hazards to both the nurse and the patient.

A participant narrated;

“…at times, we can have fifteen patients at once, and we have two beds in the emergency room and there are times that we have to nurse patients in chairs…and this makes work very difficult” (Participant 127)

“…the stretchers, wheelchairs with the beds make the place so congested…the environment is very small and the way many patients are rushed in makes the place not conducive” (Participant 125)

***Theme 2;* Understaffing at the emergency unit**

Understaffing was a major issue expressed by all participants. According to the participants, adequate staffing is necessary for effective emergency care delivery and will reduce work stress on nurses. Participants indicated that in some cases, nurses have to run shifts alone or with students amidst increasing workloads. They further emphasized that it was more worrying when clinical nurse assistants with limited scope of practice and required to work under the supervision of a

registered nurse were allowed to run shifts alone. This practice has implications on the provision of quality emergency nursing care to patients. Participants reported being overwhelmed with the increasing number of patients admitted to the unit and this affects healthcare delivery. In some cases, one nurse attends to about 6 cases per shift.

A participant narrated

> *"In terms of staff strength, we are lacking a lot to the extent that sometimes only one staff works in the emergency unit per shift"* (Participant 212)

Another participant said;

> *"…there is no trained emergency nurse in the emergency unit. Staffing challenges make work very tedious because, work that four (4) or five (5) people should do, it is left on one person. This makes nursing care very difficult and leads to exhaustion. This leads to attention deficit and inadequate care for the client. Our emergency is very busy, we work 24/7 and you could manage about twenty patients during the day shift and I am always very tired when I get home"* (Participant 127)

***Theme 3;* Lack of Emergency Equipment**

Participants expressed their views on the unavailability of logistics and consumables at the emergency unit affecting quality emergency care delivery. Due to the influx of patients to the emergency unit a two beds capacity ward is inadequate to contain the number of cases it manages. The wheelchairs and stretchers are all in a deplorable state as most are broken or worn out. Some vital resources needed for care delivery runs out of stock, and are not available at the time of need

and nurses have to use their bare hands in performing invasive procedures or wait until patients' relatives purchase some of these resources or run to other wards to borrow them. The following are narrations from some participants.

> *"…We have two BP (blood pressure) apparatuses that we share with the general Out Patient Department (OPD), so when a case comes to the emergency unit, you have to run to the OPD for the BP apparatus and the general OPD is on hold or chasing you back for the same apparatus. And the patient looks at you, that, you don't know what you are doing"* (Participant 127)

> *"Sometimes unavailability of emergency drugs brings a lot of problems in the emergency unit…we are left with nothing to work with…"* (Participant 212)

Most nurses working in the ED were left in a state of frustration as the most vital equipment needed to manage critical cases were either broken down or unavailable. A participant narrated;

> *"… sometimes patient report to the emergency and both the hospital and emergency stock is finished and we have to use our own money to buy common intravenous fluid (IVF) giving set. There are no glucometer strips to even check the patient's random blood sugar (RBS) when they come in collapsed. You either run to the wards for glucometer strips or send relative to buy them outside. We sometimes don't have gloves or syringes to work with and have to use our bare hands for most of the invasive procedures. You have situations where there is no oxygen in the whole hospital and you are found wanting in front of a client who needs oxygen..."* (Participant 311)

"… sometimes common disposable gloves are not available as well as safety boxes and it exposes us to a lot of occupational risks. You will run everywhere for oxygen just because there is no oxygen in the hospital and at the end of the day, you lose your client" (Participant 129)

***Theme 4;* Inadequate Managerial Support**

To deliver quality emergency care, overwhelmingly all participants pointed to the need to be supported and given the needed attention by management. The participants enunciated at length about the lack of support by management and how this contributes negatively to their ability to provide quality care for their clients. Some nurses singled out their unit head (in-charge) as being a supportive and motivating figure for them but seeing the nursing administration not to support their course. Most of the administrative issues were about the ineffectiveness of nursing administration- lack of effective listening and response to nurses' plight, lack of motivation, demotivating actions, and utterances. Lack of motivation from the hospital's general administration and lack of proactive effort by the hospital to continuously supply emergency drugs and other vital equipment for emergency care was a major challenge to quality emergency care delivery. All participants awaited management to validate their work-related concerns and find appropriate solutions to improve quality emergency care in the unit. Narratives below were the most pervasive among the participants

"It's very difficult to get our superiors to understand what we are facing in the emergency unit. Immediately they come and the place is busy, you will hear them say, 'this place is heavy ooo, and they will disappear'. I wish that they will come, and work with us and understand what we have been going through so that they won't be saying that we are

always complaining. This challenge is always with the nursing administration because the medical superintendent is always with us and he understands what we go through. For our nursing administration, they always say we talk or complain too much, and it has got to a time where everybody says they will do what they can, and this is affecting the quality of care given to client and job satisfaction" (Participant 127)

"…nursing administration doesn't understand the plight of we the nurses working in the emergency unit. When we complain to them about consumables and logistics, they will shout at you saying that you complain too much. Meanwhile, the client is in front of you and you can't circumvent the problem. They like using this phrase- 'you're misusing the things'. Nursing administration doesn't give us listening ears and even if they do, they will say - 'during my time it was like this'. We do what we can because our managers are not ready to support. They tag you when you are trying to push for the best interest of the client" (Participant 311)

"Over the years nobody had recognized me for the works I've been doing- not even a handshake. If you do a good job, you need to be recognized and appreciated but that never happens but they're very quick to chastise you for a little mistake done. This doesn't motivate me to give my all" (Participant 121)

Most participants after working in the emergency unit for many years believe that the hospital has no plans for nurses to go for further training in emergency care nursing, and to them, this will enhance their knowledge and skill in caring for emergency cases. They bemoaned that currently, the emergency unit has no trained emergency nurse specialist which is a major concern for all

participants. Lack of continuing professional education was seen to be endangering the future of emergency nursing practice in the hospital.

> *"… I'm not aware of any policy of the hospital concerning the upgrading of nurses and nurses working at the emergency unit. Sometimes colleague nurses may do presentations on conditions and whoever is asked to do this presentation, research, and come and present. This is not effective; we need specialist training in emergency care nursing…"* (Participant 129)

Discussion

The findings of this study highlight various forms of challenges confronting nurses working in the emergency unit. Amid these challenges, emergency department (ED) nurses continued to deliver emergency care whiles calling for their concerns to be heard and addressed to enhance quality emergency care delivery.

Overcrowding in the emergency unit was one of the major challenges expressed by nurses working in the ED, making healthcare delivery tedious a[illegible]nbearable. Nurses could not perform the full iterative circle of the nursing process due to the unconducive working environment and this affects the quality of emergency care delivery leading to poor patient outcomes. The current study finding is consistent with previous studies conducted across the globe identifying overcrowding in the emergency department as a major challenge to quality emergency care delivery [22, 23, 24, 25, 26, 27, 28, 29]. Overcrowding in the ED may occur as a result of the shortage of nursing staff, the number of patients waiting to be seen, bed shortage, delays in treating or assessing patients already in the ED, or impedi[illegible]nts to treated patients leaving the ED [22, 28, 30, 31]. Therefore, if the global crisis [32] of ED crowding is to be solved, interventions designed to address the problem

must be tailored towards identified specific causes. We recommend that hospital management should provide a spacious ED and also increase the bed capacity to contain the in-flux of patients. Also, the ED should further strengthen their triaging system by providing a triaging room at the ED as this will help identify acutely ill patients needing emergency care from non-critically ill patients who could be referred to the general ward. This will further prevent delays in referring and discharging of patients from the ED to ease crowding.

The shortage of nursing staff in the emergency department remains an important issue of interest to hospital administrators, nurses, and physicians. The current study revealed that understaffing was a challenge experienced by ED nurses. According to the participants, adequate staffing is necessary for effective emergency care delivery and will improve key ED throughput metrics. To help breach the gap most participants had to work overtime to assist their colleagues in the next shift. The findings of this study are not different from a study conducted in Canada by Enns and Sawatzky [33] where participants expressed a shortage of nursing staff in the ED which led to ED nurses working overtime. A study by Lam, Kwong Hung, Pang, and Chien [34] in China also found that a shortage of emergency nursing staff was a challenge in the ED. The World Health Report in 2006 classified Ghana among 36 countries in SSA facing health workforce crisis [35] which necessitated government interventions through the Ministry of Health to address a myriad of health workforce challenges especially nurses and midwives. Recently out of about 115,650 employed public sector health workers 58% were nurses and midwives [36, 37]. From 2008 to 2018 there has been a remarkable increase in the nursing workforce of about 370% [36]. Nonetheless, empirically, there exists a lingering shortage of nurses and midwives in most hospitals and clinics across the country [37, 38]. As in recent times over 40,000 trained nurses and

midwives have been unemployed between 2016 to 2019 whiles health facilities across the country are in dire need of these professionals. Due to the extended credit facility agreement between the government of Ghana and the International Monetary Fund with its associated austerity measures, most of the graduated nurses from 2016 and 2018 could not be immediately employed [38]. We recommend that in developing a new human resource policy and strategy for the health workforce, it would be imperative for the Ministry of Health (MOH) to embark on a comprehensive health labour market analysis paying attention to the nursing labour market dynamics to generate robust evidence to support need-based training of nurses (emergency nurses) which will meet the population health needs and avert this chronic shortage of nursing staff in the country.

Nurses in the ED recounted the challenges they encounter in delivering emergency care amid the unavailability of basic emergency equipment. In most emergency units in Ghana, patients reporting to the units are not triaged and most emergency centres are poorly equipped, under-resourced, and overcrowded [11]. Our study finding is consistent with a study conducted in Ghana by Atakro et al., [22] where nurses complained of lack of material resources to work with. Another study by Hines, Fraze, and Stocks [39] also found that ED nurses were confronted with several challenges which included inadequate resources to work with due to decreased reimbursement by insurers. Participants in the current study expressed their dissatisfaction when they lack adequate resources to work with. Currently, the provision of high-quality emergency care nurses in Ghana are an underdeveloped resource and can be improved with support from the government, hospital management, and NGOs [11]. As the ED is most often the first point of call for patients, resourcing it has an implication for quality care, quick recovery and discharge, client and relative satisfaction, good hospital image, and reducing overcrowding.

In order to deliver quality emergency care, overwhelmingly all participants pointed to the need to be supported and given the needed attention by management. The participants enunciated at length about management's lack of support and how this contributes negatively on their ability to provide quality care for their clients. This finding is consistent with a study conducted in Canada by Enns and Sawatzky [33]. It was also observed in both studies that participants yearned for management to validate their work-related concerns and find appropriate solutions to improve quality emergency care in the unit.

Nurses in Ghana are trained in emergency care largely as general nurses with limited emergency care knowledge and skills. Participants in the current study have inadequate specialist training in emergency nursing. Participants cautioned that lack of professional continuing education in emergency nursing for nurses in the unit will endanger the future of emergency nursing practice in the hospital and the country as a whole. It is therefore imperative to develop career path or progression policies that reward increasing clinical competency, knowledge, education, and professional development for nurses.

Limitations

The findings of this study are limited to the participants interviewed and to their challenges experienced in the emergency unit. Nonetheless, the rich exploration of the challenges affecting emergency care provides an opportunity for stakeholders to find solutions to address these concerns raised by ED nurses to improve quality emergency care.

Conclusion

The study identified several major challenges confronting nurses working in the emergency unit such as overcrowding, understaffing, lack of resources, and limited managerial support. These challenges if not managed and resolved will endanger quality emergency nursing care delivery.

It is important for the government, and hospital management to invest attention and resources into EDs to reduce the situation of lack of resources for the emergency team to work with. There is a need for hospital/ED managers to develop innovative strategies and policies that will support the working environment of ED nurses to provide quality emergency care for patients. Management that validates the expressions of concern from ED nurses will foster a positive working environment.

Acknowledgment

The authors are grateful to the participants of this study.

Consent to publish

Not applicable

Availability of data and materials

Data Availability Statement: All relevant data are within the manuscript and its Supporting Information files.

Conflicts of Interests

The authors declare that there are no conflicts of interest regarding the publication of this paper.

Funding

No funding for this study

Authors contribution

Conceptualization: Agani Afaya, George Kwame Kpodo, Denis Albanus Nangsire Zinle, Daniel Kofi Tayuu, Stella Asantewaa.

Data curation: Agani Afaya, George Kwame Kpodo, Robert Alhassan Kaba, Denis Albanus Nangsire Zinle, Daniel Kofi Tayuu, Stella Asantewaa.

Formal analysis: Agani Afaya, Richard Adongo Afaya, George Kwame Kpodo, Robert Alhassan Kaba, Denis Albanus Nangsire Zinle, Daniel Kofi Tayuu, Stella Asantewaa

Funding acquisition: No funding

Methodology: Agani Afaya, George Kwame Kpodo, Denis Albanus Nangsire Zinle, Daniel Kofi Tayuu, Stella Asantewaa.

Resources: Agani Afaya, Agani Afaya, George Kwame Kpodo, Denis Albanus Nangsire Zinle, Daniel Kofi Tayuu, Stella Asantewaa.

Supervision: Agani Afaya, Agani Afaya, Victoria Bam, Thomas Bavo Azongo, Richard Adongo Afaya, George Kwame Kpodo, Robert Alhassan Kaba, Denis Albanus Nangsire Zinle, Daniel Kofi Tayuu, Stella Asantewaa, Peter Adatara

Validation: Agani Afaya, Agani Afaya, Agani Afaya, Victoria Bam, Thomas Bavo Azongo, Richard Adongo Afaya, George Kwame Kpodo, Robert Alhassan Kaba, Denis Albanus Nangsire Zinle, Daniel Kofi Tayuu, Stella Asantewaa, Peter Adatara

Writing – original draft: Agani Afaya, Richard Adongo Afaya, George Kwame Kpodo, Denis Albanus Nangsire Zinle, Daniel Kofi Tayuu, Stella Asantewaa.

Writing – review & editing: Agani Afaya, Victoria Bam, Thomas Bavo Azongo, Richard Adongo Afaya, George Kwame Kpodo, Robert Alhassan Kaba, Denis Albanus Nangsire Zinle, Daniel Kofi Tayuu, Stella Asantewaa, Peter Adatara

References

1. Japiong KB, Asiamah G, Owusu-Dabo E, Donkor P, Stewart B, Ebel BE, Mock CN. Availability of resources for emergency care at a second-level hospital in Ghana: a mixed methods assessment. African Journal of Emergency Medicine. 2016;6(1):30-7.
2. Bell SA, Oteng R, Redman R, Lapham J, Bam V, Dzomeku V, Yakubu J, Tagoe N, Donkor P. Development of an emergency nursing training curriculum in Ghana. International emergency nursing. 2014;22(4):202-7.
3. Wong EG, Gupta S, Deckelbaum DL, Razek T, Kushner AL. Prioritizing injury care: a review of trauma capacity in low and middle-income countries. journal of surgical research. 2015;193(1):217-22.
4. Mock C, Quansah R, Kobusingye O, Goosen J. Trauma care in Africa: the way forward. African Journal of Trauma. 2014;3(1):3.
5. Wolf L, Brysiewicz P, LoBue N, Heyns T, Bell SA, Coetzee I, Papa A, Augustyn J, Van Eeden I, Sepeku A, Pho A. Developing a framework for emergency nursing practice in Africa. African Journal of Emergency Medicine. 2012;2(4):174-81.
6. Gosselin RA, Spiegel DA, Coughlin R, Zirkle LG. Injuries: the neglected burden in developing countries. Bulletin of the World Health Organization. 2009;87:246-a.
7. Bell SA, Bam V, Acheampong E. Developments in emergency nursing education in Ghana. Emergency Nurse. 2015;23(8).
8. Bergman S, Deckelbaum D, Lett R, Haas B, Demyttenaere S, Munthali V, Mbembati N, Museru L, Razek T. Assessing the impact of the trauma team training program in Tanzania. Journal of Trauma and Acute Care Surgery. 2008;65(4):879-83.

9. Hardcastle TC, Oteng R. Trauma care in Africa: triumphs and challenges. African Journal of Emergency Medicine. 2011;2(1):53-4.
10. Son NT, Mock C. Improvements in trauma care capabilities in Vietnam through use of the WHO-IATSIC Guidelines for Essential Trauma Care. Int J Inj Contr Saf Promot 2006;13(2):125–7.
11. Atakro CA, Gross J, Sarpong T, Armah E, Akuoko CP. Qualitative inquiry into Registered General Nurses' experiences in the emergency centre. African Journal of Emergency Medicine. 2018; 8(1):16-20.
12. Charmaz K. Constructing grounded theory. A practical guide through qualitative analysis. 2nd ed. London: Sage publications; 2007
13. Elliott R, Timulak L. Descriptive and interpretive approaches to qualitative research. A handbook of research methods for clinical and health psychology. 2005;1(7):147-59.
14. Mills J, Bonner A, Francis K. The Development of Constructivist Grounded Theory. International Journal of Qualitative Methods 2006, 5(1), 25–35.
15. Patton MQ. Qualitative research and evaluation methods. 3rd Sage Publications; Thousand Oaks, CA: 2002.
16. Cresswell JW, Plano Clark VL. Designing and conducting mixed method research. 2nd Sage; Thousand Oaks, CA: 2011.
17. Bernard HR. Research methods in anthropology: Qualitative and quantitative approaches. 3rd Alta Mira Press; Walnut Creek, CA: 2002.
18. Palinkas LA, Horwitz SM, Green CA, Wisdom JP, Duan N, Hoagwood K. Purposeful sampling for qualitative data collection and analysis in mixed method implementation

research. Administration and policy in mental health and mental health services research. 2015;42(5):533-44.

19. Padgett DK. Qualitative and mixed methods in public health. USA: SAGE publications; 2011.

20. Patton MQ, Guba EG, Lincoln YS. Effective Evaluation: Improving the Usefulness of Evaluation Results through Responsive and Naturalistic Approaches. The Journal of Higher Education. 2006

21. Tong A, Sainbury P, Craiq J. Consolidated criteria for reporting qualitative research (COREQ): a 32 item checklist for interviews and focus groups. Int J Qual Health Care. 2007;19(6):349–57.

22. Atakro CA, Ninnoni JP, Adatara P, Gross J, Agbavor M. Qualitative inquiry into challenges experienced by registered general nurses in the emergency department: a study of selected hospitals in the Volta Region of Ghana. Emergency medicine international. 2016;2016. Article ID 6082105, 7 pages

23. Robinson KS, Jagim MM, Ray CE. Nursing workforce issues and trends affecting emergency departments. Nursing Management. 2005;36(9):46-53.

24. Vasli P, Dehghan- Nayeri N. Emergency nurses' experience of crisis: A qualitative study. Japan journal of nursing science. 2016;13(1):55-64.

25. Coughlan M, Corry M. The experiences of patients and relatives/significant others of overcrowding in accident and emergency in Ireland: A qualitative descriptive study. Accident and Emergency nursing. 2007;15(4):201-9.

26. Abir M, Goldstick JE, Malsberger R, Williams A, Bauhoff S, Parekh VI, Kronick S, Desmond JS. Evaluating the impact of emergency department crowding on disposition

patterns and outcomes of discharged patients. International journal of emergency medicine. 2019;12(1):4.

27. Sun BC, Hsia RY, Weiss RE, Zingmond D, Liang LJ, Han W, McCreath H, Asch SM. Effect of emergency department crowding on outcomes of admitted patients. Annals of emergency medicine. 2013;61(6):605-11.

28. Morley C, Unwin M, Peterson GM, Stankovich J, Kinsman L. Emergency department crowding: a systematic review of causes, consequences and solutions. PloS one. 2018;13(8).

29. Trzeciak S, Rivers EP. Emergency department overcrowding in the United States: an emerging threat to patient safety and public health. Emergency medicine journal. 2003;20(5):402-5.

30. Derlet RW, Richards JR. Emergency department overcrowding in florida, new york, and texas. Southern medical journal. 2002;95(8):846-50.

31. Asplin BR, Magid DJ, Rhodes KV, Solberg LI, Lurie N, Camargo CA Jr. A conceptual model of emergency department crowding. Ann Emerg Med. 2003;42(2):173–80.

32. Hoot NR, Aronsky D. Systematic review of emergency department crowding: causes, effects, and solutions. Ann Emerg Med. 2008;52(2):126–36.

33. Enns CL, Sawatzky JA. Emergency nurses' perspectives: Factors affecting caring. Journal of Emergency Nursing. 2016;42(3):240-5.

34. Lam SK, Kwong EW, Hung MS, Pang SM, Chien WT. A qualitative descriptive study of the contextual factors influencing the practice of emergency nurses in managing emerging infectious diseases. International journal of qualitative studies on health and well-being. 2019;14(1):1626179.

35. WHO. The World Health Report: 2006: working together for health. 2006; Available from: http://apps.who.int/iris/handle/10665/43432.
36. GHS. Human resource annual report - 2018. Accra: Ghana Health Service; 2019.
37. Asamani JA, Amertil NP, Ismaila H, Francis AA, Chebere MM, Nabyonga-Orem J. Nurses and midwives demographic shift in Ghana—the policy implications of a looming crisis. Human resources for health. 2019;17(1):32.
38. Asamani JA, Amertil NP, Ismaila H, Akugri FA, Nabyonga-Orem J. The imperative of evidence-based health workforce planning and implementation: lessons from nurses and midwives' unemployment crisis in Ghana. Human Resources for Health. 2020;18(1):1-6.
39. Hines A, Fraze T, Stocks C. Emergency department visits in rural and non-rural community hospitals. Rockville: Agency for Healthcare Research and Quality; 2011
