# Supplementary material for: “We are left with nothing to work with”; challenges of nurses working in the emergency unit at a secondary referral hospital: A descriptive qualitative study
Source: PLoS One. 2021 Feb 18;16(2):e0247062. doi: 10.1371/journal.pone.0247062 (PMC7891734; doi:10.1371/journal.pone.0247062)
Supplement: Attachment — Submitted filename: PLOS ONE review.docx [file pone.0247062.s003.docx]

PLOS ONE

"“We are left with nothing to work with”; challenges of nurses working in the emergency unit at a secondary referral hospital: a descriptive qualitative study"

The manuscript highlighted the challenges experienced by nurses working in an emergence unit in Ghana. This manuscript displays knowledge of the methodical aspects of qualitative research, i.e. research design, sampling and data collection, data analysis and qualitative rigor and ethics. The participants’ voices form the themes which are further discussed in the discussion section.

My only comment is when was this study conducted? Was this before or during the pandemic that we are currently experiencing? My opinion is that if the study was conducted at the beginning/during the pandemic then the participants’ responses could be different or have more to add regarding the overcrowding and lack of resources and managerial support.
